# Genetic Findings in Short Turkish Children Born to Consanguineous Parents

**DOI:** 10.1159/000539696

**Published:** 2024-06-05

**Authors:** Sjoerd D. Joustra, Emregul Isik, Jan M. Wit, Gonul Catli, Ahmet Anik, Belma Haliloglu, Nurgun Kandemir, Elif Ozsu, Yvonne M.C. Hendriks, Christiaan de Bruin, Sarina G. Kant, Angel Campos-Barros, Rachel C. Challis, David Parry, Margaret E. Harley, Andrew Jackson, Monique Losekoot, Hermine A. van Duyvenvoorde

**Affiliations:** a Department of Paediatrics, Division of Pediatric Endocrinology, Willem-Alexander Children’s Hospital, Leiden University Medical Center, Leiden, The Netherlands; b Department of Paediatrics, Ankara Bilkent City Hospital, Ankara, Turkey; c Department of Paediatric Endocrinology, Izmir Katip Celebi University Faculty of Medicine, Izmir, Turkey; d Department of Paediatric Endocrinology, Istinye University Faculty of Medicine, Istanbul, Turkey; e Department of Paediatric Endocrinology, Dokuz Eylul University, Izmir, Turkey; f Department of Paediatric Endocrinology and Diabetology, Marmara University School of Medicine, Istanbul, Turkey; g Department of Paediatric Endocrinology, Hacettepe University, Faculty of Medicine, Ankara, Turkey; h Department of Paediatric Endocrinology and Diabetes, University of Ankara, Ankara, Turkey; i Department of Clinical Genetics, Leiden University Medical Centre, Leiden, The Netherlands; j Department of Clinical Genetics, Erasmus Medical Centre, Rotterdam, The Netherlands; k Institute of Medical and Molecular Genetics (INGEMM), IdiPAZ, Hospital Universitario La Paz, Madrid, Spain; l Rare Diseases Biomedical Research Network (CIBERER; U 753), ISCIII, Madrid, Spain; m MRC Human Genetics Unit, Institute of Genetics and Cancer, University of Edinburgh, Edinburgh, UK

**Keywords:** Short stature, Consanguinity, Single nucleotide variants, Copy number variants, Growth hormone

## Abstract

**Introduction:**

The diagnostic yield of genetic analysis in the evaluation of children with short stature depends on associated clinical characteristics, but the additional effect of parental consanguinity has not been well documented.

**Methods:**

This observational case series of 42 short children from 34 consanguineous families was collected by six referral centres of paediatric endocrinology (inclusion criteria: short stature and parental consanguinity). In 18 patients (12 families, group 1), the clinical features suggested a specific genetic defect in the growth hormone (GH) insulin-like growth factor I (IGF-I) axis, and a candidate gene approach was used. In others (group 2), a hypothesis-free approach was chosen (gene panels, microarray analysis, and whole exome sequencing) and further subdivided into 11 patients with severe short stature (height <–3.5 standard deviation score [SDS]) and microcephaly (head circumference <−3.0 SDS) (group 2a), 10 patients with syndromic short stature (group 2b), and 3 patients with nonspecific isolated GH deficiency (group 2c).

**Results:**

In all 12 families from group 1, (likely) pathogenic variants were identified in *GHR, IGFALS, GH1*, and *STAT5B*. In 9/12 families from group 2a, variants were detected in *PCNT, SMARCAL1, SRCAP, WDR4*, and *GHSR*. In 5/9 families from group 2b, variants were found in *TTC37, SCUBE3, NSD2, RABGAP1*, and 17p13.3 micro-deletions. In group 2c, no genetic cause was found. Homozygous, compound heterozygous, and heterozygous variants were found in 21, 1, and 4 patients, respectively.

**Conclusion:**

Genetic testing in short children from consanguineous parents has a high diagnostic yield, especially in cases of severe GH deficiency or insensitivity, microcephaly, and syndromic short stature.

## Introduction

The diagnostic approach of a child presenting with short stature, defined as a height standard deviation score (SDS) below −2 and/or decreased growth velocity, has changed considerably over the last two decades. Traditionally, the focus was on detecting dysmorphic syndromes or disorders of the endocrine or other organ systems. In most cases, no definite diagnosis could be made, so that the attribution of descriptive diagnostic labels like “children born small for gestational age with failure of catch-up growth” or “idiopathic short stature,” which includes familial idiopathic short stature and constitutional delay of growth and puberty [1], would often mark the end of the diagnostic workup [2]. In the last two decades, many new genetic tools have become available, which has led to the discovery of numerous novel genetic defects associated with short stature [3–13]. It has also become clear that the prevalence of monogenic growth disorders is much higher than previously assumed, and that their phenotypic variability is substantial [8, 13, 14].

In some cases, the clinical and biochemical features of a short patient are so specific or indicative for a certain condition that the clinician can use the traditional candidate-gene approach. However, a gene panel or an exome-/genome-wide approach is often more successful and cost-effective [9, 11]. Until recently, this hypothesis-free approach consisted of consecutive analysis of copy number variants (CNVs) through microarrays and pathogenic single nucleotide variants through massive parallel sequencing using a gene panel or whole exome sequencing (WES). Present technology has made it possible to perform both single nucleotide variants and CNV analysis on massive parallel sequencing data [15, 16].

The current challenge is to decide which children with short stature should be tested for genetic causes. Several clues from the clinical assessment can increase the pretest likelihood of a monogenic defect or causal CNV [3, 17]. The diagnostic yield is relatively high if the short child presents with additional clinical features, such as (facial) dysmorphisms, body disproportion, congenital anomalies, neurodevelopmental disorders, microcephaly or relative macrocephaly, signs of skeletal dysplasia, and severe short stature [3, 8, 10, 12, 13, 17, 18]. Abnormal biochemical findings can also suggest a monogenic defect, such as in patients with severe growth hormone (GH) deficiency or insensitivity [13, 19, 20]. Furthermore, an adequate assessment of the family history is important. A dominant pattern of inheritance is suggestive for hap-loinsufficiency of a gene involved in growth plate biology, such as *IGF1R, SHOX, NPR2, ACAN, NPPC, IHH*, and genes associated with collagenopathies [3, 6, 14, 17, 21–23]. In contrast, in the offspring of consanguineous parents, the likelihood of a recessive condition is expected to be increased. The purpose of the current paper was to describe the yield of extensive genetic testing in diagnosing the cause of short stature in 42 children from 34 consanguineous families.

## Materials and Methods

### Subjects

**Fig. 1:**
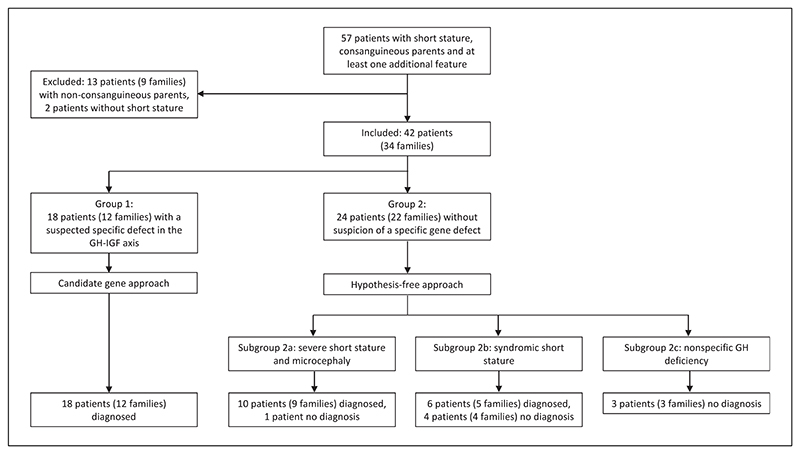
Diagram showing the selection of short patients with consanguineous parents who were investigated and the results.

Out of a total of 57 patients from Turkish referral centres of paediatric endocrinology, we included 42 patients (from 34 families) in this study who complied with the inclusion criteria: age below 18 years, short stature, parental consanguinity, and at least one additional feature associated with increased risk for a genetic defect (Fig. 1), e.g., GH deficiency, height <−3.5 SDS, microcephaly, or syndromic features. These patients were identified in six Turkish paediatric endocrinology centres and discussed with the Leiden Genetics of Growth Expertise Center of the Leiden University Medical Center (LUMC) in the Netherlands before DNA was submitted.

Data on weight, length, and head circumference (HC) at birth, height, body mass index (BMI), and sitting height/height were expressed as SDS for age and sex based on Turkish reference data [24, 25]. HC was expressed as SDS for Dutch children [26]. Serum IGF-I and IGFBP-3 were expressed as SDS for local standards. The GH peak at GH stimulation (GHmax) was expressed in ng/mL. In 9 patients, an IGF-I generation test was performed, according to local protocols.

Patients were divided into two groups. Group 1 consisted of patients in whom the clinical features suggested a specific genetic defect in the GH-IGF-I axis, so that a candidate gene approach was chosen. These DNA samples were investigated in the Laboratory for Diagnostic Genome analysis in the LUMC (Leiden, the Netherlands). Group 2 consisted of short patients with at least one additional clinical feature and tested with a hypothesis-free approach. This group was further subdivided into three subgroups. Subgroup 2a included patients with severe short stature (height <−3.5 SDS) and microcephaly (HC <−3.0 SDS). Most of these were investigated in the MRC Human Genetics Unit of the University of Edinburgh in the United Kingdom with a gene panel (see online suppl. data; for all online suppl. material, see https://doi.org/10.1159/000539696). Subgroup 2b included non-GH-deficient subjects with syndromic short stature, without clues for a specific clinical diagnosis. They were analysed in the LUMC in a step-wise approach using SNP array, custom gene panels using human phenotype ontology terms, and exome-wide analysis of WES data of the patient and both parents (trio WES). Subgroup 2c consisted of 3 patients with isolated GH deficiency in whom no specific gene defect was apparent at clinical evaluation (“nonspecific GH deficiency”). Their DNA was sent to the Molecular Endocrinology Division of the Institute of Medical and Molecular Genetics at the La Paz University Hospital in Madrid (Spain) for trio WES and CNV analysis.

### Genetic Diagnostic Procedures

Genomic analyses were performed using DNA samples obtained from leukocytes. The candidate gene approach was performed using Sanger sequencing and multiplex ligation-dependent probe amplification. Target enrichment kits, sequencing platforms, and WES statistics are reported in the online supplemental data. Data processing, variant filtering, and classification were performed using laboratory-specific standard procedures (see online suppl. data). Co-segregation analysis was performed by Sanger sequencing in some families (primer sequences available on request).

## Results

In group 1, the candidate gene approach led to a genetic diagnosis in all 12 families (18 patients) (Table 1). In group 2, a genetic diagnosis was established in 14 out of 22 families (64%) (16 out of 24 patients; 67%). In subgroup 2a, the genetic cause was established in nine out of ten families, and in subgroup 2b in five out of nine families (Table 2). In the 3 patients with nonspecific isolated GH deficiency (subgroup 2c), no pathogenic variants that could explain the phenotype were identified. The majority of genetic defects were homozygous (21/26), one was compound heterozygous and four were heterozygous.

### Group 1: Subjects Suspected of a Specific Genetic Defect in the GH-IGF Axis

Clinical and genetic results are presented in online supplementary Table S1 and Table 1, respectively. In two siblings in family 1 with the classical biochemical presentation of GH-deficiency type 1A and neonatal hypoglycaemia [27], a previously described *GH1* deletion of exons 3–5 was found by Multiplex Ligation-dependent Probe Amplification [28]. Remarkably, in the younger sibling, microcephaly was noted. Treatment with recombinant human GH (rhGH) was initiated, but the patients were lost to follow-up.

In 9 patients suspected for Laron syndrome from five unrelated families (families 2–6), four (likely) pathogenic *GHR* variants were found. Families 2 and 3 live in the same city and carry the same p.Asn115Thr variant that was previously identified in Saudi Arabia [29]. The families were unaware of common inheritance. The novel p.Val303Asp variant in family 4 is located in the highly conserved C-terminal domain of the protein and was assigned likely pathogenic. The p.Ser58Leu variant in family 5 has been described previously [30]. All patients had very low serum levels of IGF-I and IGFBP-3, but baseline serum GH was elevated in only 3 patients. In families 2, 3, 4 and 6, mean height SDS was −3.4 (range −4.3 to −2.1, *n* = 7), mean HC −1.55 (range −2.5 to −1.1, *n* = 6), and HC SDS minus height SDS 1.6 (range −0.1–3.3, *n* = 6). The two siblings in family 5 had far more severe short stature (height −7.2 and −11.2 SDS, HC unavailable).

Patient 6 showed a pseudoexon inclusion in *GHR* caused by a homozygous deep-intronic variant (c.618 + 792A>G), consistent with a reportedly relatively mild form of Laron syndrome [31]. His sensorineural deafness was caused by a homozygous pathogenic nonsense variant in *ADGRV1* (NM_032119.4, c.7446C>G, p.Tyr2482*) confirming the diagnosis of Usher syndrome type 2C (MIM #605472). His hypothyroidism may be associated with the heterozygous variant of unknown significance in *DUOX1* (NM_175940.3, c.2036G>A, p.Arg679His.

Patient 7 presented at the age of 17 years with short stature, ichthyosis, midface hypoplasia, frontal bossing, and hyperprolactinemia, suggestive for a bi-allelic *STAT5B* variant, despite the absence of any history of frequent infections or lung problems. She showed a homozygous novel truncating variant in *STAT5B*. Further clinical details of this patient were recently reported [32]. She also presented with normogonadotrophic primary amenorrhoea at Tanner 4, which has not been observed in previously reported patients. We postulate that this may be an additional clinical feature of this condition, possibly related to her hyperprolactinemia (225 ng/mL, reference range 2.5–25 ng/mL).

Offspring of families 8–12 were diagnosed with acid-labile subunit deficiency caused by four likely pathogenic homozygous or compound heterozygous *IGFALS* variants. Clinical details of these patients and their relatives were reported previously [33].

### Subgroup 2a: Subjects with Severe Short Stature (Height <−3.5 SDS) and Microcephaly (HC <−3.0 SDS)

Eleven patients from 10 families presented with severe short stature and microcephaly. A summary of the clinical data is presented in online supplementary Table S2 and in the clinical information. Genetic results are shown in Table 2.

Patients 13 and 14 from two reportedly unrelated families living in the same city were homozygous for an identical pathogenic variant in *SMARCAL1*, consistent with the diagnosis of Schimke immuno-osseous dysplasia (MIM #242900). The variant was previously reported [34–38]. In patient 15, a previously reported heterozygous pathogenic *SRCAP* variant was detected [39–44], consistent with Floating-Harbor syndrome (MIM #136140).

In 5 patients from families 16–19, homozygosity was shown for four different (likely) pathogenic *PCNT* variants, with the typical clinical features of microcephalic osteodysplastic primordial dwarfism type 2 (MOPD2, MIM #210720). The variants in patients 16, 18, and 19 are novel, while the variant in family 17 has been reported previously in 2 Turkish patients [45] and elsewhere [46, 47].

In patient 20, a novel homozygous *WDR4* variant was detected. This gene encodes a tRNA methyltransferase, and homozygous loss of function is associated with primordial dwarfism (“microcephaly, growth deficiency, seizures, and brain malformations,” MIGSB, MIM #618346) [13, 48–52].

In patient 21, we detected a heterozygous variant of unknown significance in *GHSR*, which was also present in his short mother and sister, and not in the normal-statured father, brother, and sister. The phenotype and chemotype of patients carrying *GHSR* variants are diverse, including normal or delayed pubertal development and normal or low GHmax in GH stimulation tests [20]. Although limited information is available about the Tanner stages of our patient, delayed puberty is very likely based on the shape of the growth curve, considerable bone age delay (2.7 years at discontinuation of 9 months of rhGH treatment), and an increase in height SDS in late adolescence resulting in an adult height of −2.0 SDS. In patient 22, no genetic abnormality was detected in any gene associated with primordial dwarfism, nor in chromosomal microarray analysis.

### Subgroup 2b: Patients with Syndromic Short Stature

Ten patients from 9 families presented with syndromic short stature. A summary of the clinical data is presented in online supplementary Table S3 and in the clinical information. Genetic results are shown in Table 2.

In patient 23, born extremely small for gestational age, SNP-array analysis showed two novel 17p13.3 microdeletions separated by a small non-deleted region of 101.5 kb. These heterozygous deletions were not found in the mother’s DNA (the father’s DNA was unavailable). The terminal deletion (minimal size 1.9 Mb, 1,984 probes, from 525 bp to 1,922,715 bp) contains 31 protein-coding genes including *YWHAE* (also known as 14-3-3 epsilon) and *CRK*. The interstitial deletion (minimal size 238.5 kb, 420 probes, from 2,024,217 bp to 2,262,703 bp) contains 4 protein-coding genes (*SMG6, SGSM2, SRR*, and *TSR1*). Both deletions have not been described as genomic variants in the population. The deletions do not contain the LIS1 gene (*PAFAH1B1*). The short arm of chromosome 17 is characterized by a high density of low copy repeats, creating the opportunity for non-allelic homologous recombination to occur. There are three classes of contiguous gene deletion syndromes known in this region: (1) isolated lissencephaly sequence: *PAFAH1B1* deleted, *YWHAE* not deleted (LIS1; MIM #607432); (2) *YWHAE* and/or *CRK* deleted, *PAFAH1B1* not deleted; and (3) Miller-Dieker syndrome: both areas deleted (MIM #247200). Our patient is an example of the second class, so far observed in 19 patients [53]. Her clinical features are similar to those described for other patients with a similar genotype (for details, see online suppl. information). A recent paper showed that *YWHAE* loss-of-function variants cause a neurodevelopmental disease with brain abnormalities and that individuals with variants affecting *YWHAE* alone have milder features than those with larger deletions [54]. Since linear growth is normal in individuals with isolated *YWHAE* defects [54] and severely decreased if *CRK* is deleted [55, 56], *CRK* deletion seems responsible for the short stature observed in class 2 and 3 deletions. In patient 24, genetic analysis showed a homozygous pathogenic *TTC37* variant, reported previously [57, 58], consistent with trichohepa-toenteric syndrome 1 (MIM #222470).

In the two siblings in family 25, we discovered a homozygous novel *SCUBE3* variant, which at that time had not been described as a cause of short stature. In an international research project, eight more families were identified, and functional studies were performed in *Scube3*–/– mice. This led to the identification of a novel genetic cause of dysmorphic short stature, now called “Short Stature, Facial Dysmorphism, And Skeletal Anomalies With Or Without Cardiac Anomalies 2” (SSFSC2, MIM #619184) [59].

In patient 26, a de novo frameshift variant in *NSD2* was detected. *NSD2* loss-of-function variants cause decreased methylation activity and are associated with a distinct developmental phenotype partially overlapping with Wolf-Hirschhorn syndrome (4p16.3 deletion syndrome) [60]. In a recent paper on a comprehensive series of 18 patients carrying heterozygous missense, elongating, or truncating *NSD2* variants, the core NSD2-associated phenotype was shown to include mostly mild developmental delay, prenatal-onset growth retardation, low BMI, and characteristic facial features distinct from Wolf-Hirschhorn syndrome. The authors proposed that NSD2 deficiency may be named Rauch-Steindl syndrome after the delineators of this phenotype [60]. Most patients display mild cognitive impairment, but some go to a regular school as did our patient. Formal IQ testing was not performed.

In patient 27, we found a novel homozygous variant in *RABGAP1*, which was recently described as the cause of a neurodevelopmental syndrome in 5 patients (carrying three different variants) with intellectual disability, microcephaly, bilateral sensorineural hearing loss, seizures, and overlapping dysmorphic features [61].

In patients 28–31, no definitive genetic diagnosis could be made. For clinical and genetic findings, see online supplementary information.

### Subgroup 2c: Subjects with Nonspecific Isolated GH Deficiency

Characteristics of the 3 patients with isolated GH deficiency are shown in online supplementary Table S4 and the clinical information. The results of the WES analysis were non-conclusive. Patient 34 presented with GH deficiency plus widespread severe eczema, onycho-mycosis, cheilitis, peeling skin, and acral punctate keratosis. His skin phenotype is likely caused by a homozygous nonsense mutation in *CAST* as revealed by duo WES analysis of the proband and his mother [62]. There is no known association between variants in *CAST* and GH deficiency or short stature.

## Discussion

We performed an extensive genetic analysis in 42 short children from 34 Turkish consanguineous families and observed a high diagnostic yield: in 26 out of 34 families (76%), a genetic cause was found (34 out of 42 short children, 81%). The candidate gene approach resulted in a genetic diagnosis in all 12 families (group 1), with pathogenic variants in several genes in the GH-IGF axis: *GH1* (1 family), *GHR* (5 families), *STAT5B* (1 family), and *IGFALS* (5 families). A gene panel in patients with microcephalic primordial dwarfism (subgroup 2a) resulted in a positive diagnosis in 9 out of 10 families. In patients with syndromic short stature (subgroup 2b), a genetic cause was found in 5 out of 9 families. In none of the 3 patients with nonspecific isolated GH deficiency (sub-group 2c), a genetic cause was found.

Regarding genetic defects of the GH-IGF axis (group 1), we show that based on clinical features, growth pattern, and laboratory investigations (serum IGF-I, IGFBP-3, prolactin, GH stimulation testing, and IGF generation test), the most likely candidate gene can be identified. Severe postnatal short stature in a child with normal birth weight, neonatal hypoglycaemia, unmeasurable serum GH before and after stimulation, and very low serum IGF-I and IGFBP-3, strongly point into the direction of a *GH1* deletion (Family 1). Most of our patients with Laron syndrome showed the classical phenotype and chemotype, but notably baseline serum GH was not elevated in most patients. In the patient with a novel homozygous *STAT5B* variant, the eczema and elevated serum prolactin in combination with the very low serum IGF-I and IGFBP-3 pointed into the direction of this disorder, but the low baseline and stimulated GH secretion and mild clinical presentation were unexpected. Patients with bi-allelic *IGFALS* variants are characterized by a modest short stature and a serum IGFBP-3 SDS that is considerably lower than IGF-I SDS [33].

The patients with confirmed Laron syndrome had an extremely variable height SDS (range −2.1 to −11.2 SDS), consistent with previous observations [63]. Two out of 6 patients were borderline microcephalic (−2.0 and −2.5 SDS), and patient 3b had a similar SDS for height and HC, in contrast to the classical presentation of Laron syndrome. A recent study exemplifies that exome sequencing in these patients might reveal additional genetic defects responsible for microcephaly [64], as consanguinity increases the likelihood of multiple recessive genetic conditions. While serum IGF-I and IGFBP-3 concentrations were extremely low in all patients, baseline serum GH concentrations were elevated in only three of them.

As expected for offspring of consanguineous couples, most patients were homozygous carriers of gene variants (21 families). However, one should note that in 4 families, the patient’s short stature was caused by a heterozygous defect and in one family by compound heterozygosity. This finding illustrates that in offspring of consanguineous marriages, pathogenic genetic aberrations may not necessarily present as homozygous variants, in line with a previous report in a Saudi cohort [65]. Our study also shows that several patients not only carry gene variants responsible for short stature but also have (homozygous) variants that explain additional clinical features. An example is patient 6 who carries homozygous variants in *GHR* as well as in *ADGRV1* (responsible for his sensorineural deafness).

Regarding our patients in whom targeted gene panels or trio WES analysis did not yield a genetic diagnosis, we speculate that these may carry variants in genes which have not been associated with short stature yet, or genetic defects outside of the coding areas of the genome. In the future, whole genome sequencing, RNA sequencing (including analysis of microRNAs and long-noncoding RNAs), or epigenetic analyses may be needed to establish the diagnosis.

Regarding the clinical benefit of genetic testing in severe growth disorders, there are at least four reasons why the identification of rare monogenic causes is beneficial [3]. First, the identification of a molecular aetiology can end the diagnostic workup for the patient and provide the family with an answer as to why their child is not growing normally. Second, the genetic diagnosis may alert the clinician to medical comorbidities for which the patient is at risk. This does not only benefit the patient but may also alert the affected relatives to such comorbidities, for example, early-onset osteoarthritis and degenerative disc disease if an *ACAN* variant is identified [66]. Third, the determination of a molecular aetiology is invaluable for genetic counselling. Fourth, the genetic aetiology may have implications for therapy, in particular, whether rhGH treatment may be efficacious and safe. For the patients with disorders of the GH-IGF axis, there are clear therapeutic consequences: rhGH treatment for those with a *GH1* or *GHSR* defect, recombinant human IGF-I (rhIGF-I) treatment for those homozygous for *GHR* or *STAT5B* variants, and a contraindication of rhGH or rhIGF-I in those with bi-allelic *IGFALS* variants because of the expected poor growth response [67].

We suggest two more reasons why the identification of rare monogenic causes can be beneficial. First, increasing the number of patients with short stature who undergo genetic testing will lead to a better insight into the broadness of the spectrum of clinical phenotypes associated with genetic syndromes, which will in turn help the identification of future cases. An example from our patients is the elaborate description of the phenotype of patients carrying bi-allelic *IGFALS* variants [33]. Another example is the observation that short stature is a clinical feature associated with the recently described syndrome associated with bi-allelic *RABGAP1* variants [61]. Second, genetic testing can lead to the uncovering of novel syndromes, such as the syndrome caused by bi-allelic *SCUBE3* variants which we discovered in the two affected siblings in family 24, and reported jointly with other investigators [59].

There is also a potential benefit of describing the clinical features of patients in whom genetic findings are still uncertain, for example, the *LARP7* variant found in patient 30 and the *SPATA5* variant we detected in patient 31 (online suppl. information). If in the future other patients with a similar phenotype are found who carry a variant in one of these genes, this may lead to the identification of a novel genetic cause of short stature. Lastly, we would like to emphasize that the observed frequency of genetic causes of short stature is dependent on the population that is studied and likely differs between patients from different ancestries.

In conclusion, thorough genetic analysis in short children from consanguineous parents has a high diagnostic yield, especially in case of severe GH deficiency and insensitivity, microcephaly, and syndromic short stature. Diagnosing these patients has important clinical consequences and provides more insights into the scope of the clinical features associated with monogenic causes of short stature. While most patients carried homozygous genetic defects, heterozygous and compound heterozygous defects were also found.

## Supplementary Material

Supplementary material

## Figures and Tables

**Table 1:** Group 1: genetic diagnosis in patients suspected of a specific genetic defect in the GH-IGF axis using a candidate gene approach

Family No.	Cases, *n*	Gene	Inheritance	Genomic variant^1^	cDNA^1^	Protein^1^	Classification *ACMG category*	Diagnosis, MIM No.
1	2	*GH1*	Hom	NC_000017.10:g.(? _61995432) _(61994614 _?)del	NM_000515.3 Deletion exon 3, 4, 5	Truncated protein/no protein expressed	Pathogenic *PSV1, PM3*	IGHD1A #262400
2	1	*GHR*	Hom	Chr5(GRCh37): g.42695096A>C	NM_000163.4: c.344A>C	p.(Asn115Thr)	Likely pathogenic *PM1, PM2, PM3_sup, PP5*	Laron syndrome #262500
3	3	*GHR*	Hom	Chr5(GRCh37): g.42695096A>C	NM_000163.4: c.344A>C	p.(Asn115Thr)	Likely pathogenic *PM1, PM2, PM3_sup, PP5*	Laron syndrome #262500
4	2	*GHR*	Hom	Chr5(GRCh37): g.42718186T>A	NM_000163.4: c.908T>A	p.(Val303Asp)^2^	Likely pathogenic *PM2, PM3_sup, PP3, PP4_mod*	Laron syndrome #262500
5	2	*GHR*	Hom	Chr5(GRCh37): g.42689028C>T	NM_000163.4: c.173C>T	p.(Ser58Leu)	Likely pathogenic *PM2, PM3_sup,* *PP3, PP5*	Laron syndrome #262500
6	1	*GHR*	Hom	Chr5(GRCh37): g.42700896A>G	NM_000163.4: c.618+792A>G	p.?	Likely pathogenic *PS3,* *PM2,* *PM3_sup, PP5*	Laron syndrome #262500
7	1	*STAT5B*	Hom	Chr17(GRCh37): g.40368052del	NM_012448.3: c.1453delG	p.(Asp485Thrfs*29)^2^	Pathogenic *PVS1, PM2, PM3_sup*	GHISID1 #245590
8	2	*IGFALS*	Hom	Chr16(GRCh37): g.1840957C>T	NM_004970.2: c.1462G>A	p.(Asp488Asn)^2^	Likely pathogenic *PS4, PM2, PM3_sup*	ACLSD #615961
9	1	*IGFALS*	Hom	Chr16(GRCh37): g.1840957C>T	NM_004970.2: c.1462G>A	p.(Asp488Asn)^2^	Likely pathogenic *PS4, PM2, PM3_sup*	ACLSD #615961
10	1	*IGFALS*	Hom	Chr16(GRCh37): g.1842168T>C	NM_004970.2: c.251A>G	p.(Asn84Ser)^2^	Likely pathogenic *PS4,* *PM2,* *PM3_sup, PP3*	ACLSD #615961
11	1	*IGFALS*	Hom	Chr16(GRCh37): g.1840942del	NM_004970.2: c.1477del	p.(Arg493Alafs*176)^2^	Likely pathogenic *PVS1, PM2, PM3_sup*	ACLSD #615961
12	1	*IGFALS*	Comp Het	Chr16(GRCh37): g.1842168T>C g.1840957C>T	NM_004970.2: c.251A>G c.1462G>A	p.(Asn84Ser)^2 ^ p.(Asp488Asn)^2^	Likely pathogenic (both) *PM1, PM2, PM3, PP3*	ACLSD #615961

ACMG, American College of Medical Genetics and Genomics; Comp Het, compound heterozygous; Gr, Group; Het, heterozygous; Hom, homozygous; LP, likely pathogenic; P, Pathogenic; ACLSD, acid-labile subunit deficiency; GHDP, isolated partial growth hormone deficiency; GHISID1, growth hormone insensitivity syndrome with immune dysregulation 1, autosomal recessive; IGHD1A, isolated growth hormone deficiency, type Ia.

1 Human genome variation society (HGVS) nomenclature.

2 Novel variant.

**Table 2:** Genetic diagnosis in patients with microcephalic severe short stature (group 2a) and syndromic short stature (group 2b) using a hypothesis-free approach

Family (No. cases)	Gene	Inheritance	Genomic variant (HGVS)	cDNA	Protein	ACMG category	Diagnosis, MIM
Group 2a							
13	*SMARCAL1*	Hom	Chr2(GRCh37): g.217341863G>A	NM_014140.4: c.2459G>A	p.(Arg820His)	Pathogenic *PP5* *PM2 PM3 PP3*	SIOD #242900
14	*SMARCAL1*	Hom	Chr2(GRCh37): g.217341863G>A	NM_014140.4: c.2459G>A	p.(Arg820His)	Pathogenic *PP5* *PM2 PM3 PP3*	SIOD #242900
15	*SRCAP*	Het (de novo)	Chr16(GRCh37): g.30748664C>T	NM_006662.3: c.7303C>T	p.(Arg2435^*)^	Pathogenic *PP5* *PVS1 PM2 PS2*	Floating harbour #136140
16	*PCNT*	Hom	Chr21(GRCh37): g.47831167dup	NM_006031.6: c.5180dup	p.(Asn1727Lysfs*14)^1^	Likely pathogenic *PVS1 PM2*	MOPD2 #210720
17 (2)	*PCNT*	Hom	Chr21(GRCh37): g.47786998G>T	NM_006031.6: c.3109G>T	p.(Glu1037^*)^	Pathogenic *PVS1* *PM2 PP5 PM3*	MOPD2 #210720
18	*PCNT*	Hom	Chr21(GRCh37): g.47766655A>G	NM_006031.6: c.721−2A>G	p.?	Likely pathogenic *PVS1 PM2*	MOPD2 #210720
19	*PCNT*	Hom	Chr21(GRCh37): g.47809114_47809346del	NM_006031.6: c.3608_3840del	p.(Pro1204Glyfs*11)^1^	Likely pathogenic *1A 2B 2E 3A*	MOPD2 #210720
20	*WDR4*	Hom	Chr21(GRCh37): g.44283575C>T	NM_018669.6: c.428G>A	p.(Gly143Glu)^1^	Likely pathogenic *PM2 PM3_SUP PM1* *PP3_SUP*	MIGSB #618346
21	*GHSR*	Het (mat)	Chr3(GRCh37): g.172163003G>C	NM_198407.2: c.1049C>G	p.(Thr350Ser)	VUS *PM2 PPI* *PP5 BP4*	GHDP #615925
Group 2b							
23	Two 17p13.3 deletions	Het^2^ (probably de novo)	arr[hg19] 17p13.3(525−1,922,715)x1, 17p13.3(2,024,217-2,262,703)x1^1^				
24	*TTC37*	Hom	Chr5(GRCh37): g.94803618C>T	NM_014639.4: c.4572G>A	p.(Trp1524^*)^	Likely pathogenic *PM2 PVS1 PP5*	THES1 #222470
25 (2)	*SCUBE3*	Hom	Chr6(GRCh37): g.35213204T>C	NM_152753.4: c.2599+2T>C	p.?^1^	Likely pathogenic *PVS1 PM2 PM3*	SSFSC2 #619184
26	*NSD2*	Het (de novo)	Chr4(GRCh37): g.1905987dup	NM_001042424.3: c.642dup	p.(Asp215Argfs*10)^1^	Pathogenic *PVS1* *PS2 PM2*	WHS #194190
27	*RABGAP1*	Hom	Chr9(GRCh37): g.125861049dup	NM_012197.4: c.2789dup	p.(Asn930Lysfs*7)^1^	Likely pathogenic *PVS1 PM2* *M3_SUP*	*RABGAP1-* related syndrome

ACMG, American College of Medical Genetics and Genomics; Comp Het, compound heterozygous; Gr, Group; HGVS, human genome variation society; Het, heterozygous; Hom, homozygous; LP, likely pathogenic; P, pathogenic; syndr, syndrome; GHDP, isolated partial growth hormone deficiency; MDLS, Miller-Dieker lissencephaly syndrome; MIGSB, microcephaly, growth deficiency, seizures, and brain malformations; MOPD2, microcephalic osteodysplastic primordial dwarfism type 2; SIOD, Schimke immunoosseous dysplasia; SSFSC2, short sature, facial dysmorphism, and skeletal anomalies with or without cardiac anomalies 2; SSOAOD, short stature and advanced bone age, with or without early-onset osteoarthritis and/or osteochondritis dissecans; THES1, trichohepatoenteric syndrome 1; WHS, Wolf-Hirschhorn syndrome.

1 Novel variant.

2 The deletions were not found in the mother’s DNA, but the father’s DNA was not available.

## Data Availability

WES datasets have not been deposited in a public repository due to privacy and ethical restrictions but are available from the corresponding author on request.
