# Supplementary material for: Genetic Findings in Short Turkish Children Born to Consanguineous Parents
Source: Horm Res Paediatr. Author manuscript; Available in PMC 2024 Sep 20. (PMC7616538; doi:10.1159/000539696)
Supplement: Supplementary material [file EMS197877-supplement-Supplementary_material.docx]

***Supplemental Data***

**Genetic findings in short Turkish children born to consanguineous parents**

Sjoerd D. Joustra*, Emregul Isik*, Jan M. Wit*, Gonul Catli, Ahmet Anik, Belma Haliloglu, Nurgun Kandemir, Elif Ozsu, Yvonne M.C. Hendriks, Christiaan de Bruin, Sarina G. Kant, Angel Campos-Barros, Rachel C. Challis, David Parry, Margaret E. Harley, Andrew Jackson, Monique Losekoot^#^, Hermine A. van Duyvenvoorde^#^

*These authors contributed equally to this paper

#These authors share last authorship

**SUPPLEMENTAL LABORATORY INFORMATION**

In the department of Clinical Genetics in Leiden, genomic DNA was extracted from blood samples using the Chemagen automated DNA isolation work station (Janus Chemagic 360, Perkin Elmer). Analysis of candidate genes was performed using Sanger sequencing of the exons including +/- 20 nucleotides of intronic sequences and analysis of intron 6 of *GHR* was performed using standard procedures. MLPA kits P216 and P262 MRC Holland, The Netherlands were used for CNV detection. WES was performed using the Agilent SureSelectXT Human All Exome V5 or V7 kit following the manufacturer instructions in an Illumina platform, with >94% of targeted regions were covered at >= 20x depth. The exome sequencing protocol was validated for clinical use according to ISO 15189. After 'read alignment' using BWA and 'variant calling' using GATK, the annotation was performed using the in-house sequence analysis pipeline ‘Modular GATK-Based Variant Calling Pipeline (MAGPIE)’. MOON software (Invitae) was used for further HPO-based (https://hpo.jax.org/app/) analysis and interpretation of variants. The pathogenicity of variants was classified according to the ACMG/AMP criteria [1, 2]. A full list of detected variants in our patients is available on request.

In the MRC HGU in Edinburgh (United Kingdom), for patients 14-20 and 22, genomic DNA was extracted from blood samples by standard methods or from saliva samples using Oragene collection kits according to the manufacturer’s instructions. Informed consent was obtained from all participating families as a research study approved by the Scottish Multicentre Research Ethics Committee (05/MRE00/74). Mutation analysis of *PCNT* was performed by Sanger sequencing of all coding exons [3], in cases phenotypically matching MOPD II. For remaining cases, patients were screened for Microcephaly and Primordial dwarfism genes using a next generation sequencing panel. Target enrichment was performed using a custom-designed Twist baits (Twist Bioscience), with coverage of >99% of all coding exons as reported on RefSeq and their immediate intronic sequences (+/- 15bp) for 91 genes. Library preparation was performed with Nextera Flex for Enrichment reagent kit according to manufacturer’s instructions (Illumina), with 150bp paired-end sequencing on the MiSeq platform (Illumina), with >98% of targeted regions were covered at >= 20x depth. The following genes associated with primordial dwarfism, primary microcephaly and associated syndromes were analysed: *ANKLE2, ANKRD11, ARCN1, ASPM, ASXL3, ATR, ATRIP, ATRX, BLM, CASK, CDC45, CDC6, CDK5RAP2, CDKL5, CDKN1C, CDT1, CENPE, CENPF, CENPJ, CEP135, CEP152, CEP63, CIT, CREBBP, DDX11, DNA2, DNMT3A, DONSON, DPP6, DYRK1A, EED, EP300, ERCC6, ERCC8, ESCO2, FANCD2, FOXG1, GMNN, IGF1, IGF1R, KAT6B, KIF11, KMT2A, KNL1, LARP7, LIG4, MCM4, MCPH1, MED12, MRE11A, MYCN, NBN, NCAPD2, NCAPD3, NCAPH, NDE1, NSUN2, ORC1, ORC4, ORC6, PCNT, PHC1, PHGDH, PIK3R1, PLK4, PNKP, POC1A, POLA1, POLE, RAD50, RAD51, RBBP8, RBM10, RNU4ATAC, RTTN, SETD2, SMARCAL1, SRCAP, STIL, TCF4, TONSL, TOP3A, TRAIP, TUBGCP6, VPS13B, WDR4, WDR62, WHSC1, XRCC1, XRCC4, ZNF335*.

In the INGEMM in Madrid (Spain), for patients 32-34, WES of the genomic DNA of the three probands and available parent samples was performed using the Agilent SureSelect Human All Exome V6 (58M) kit following the manufacturer instructions in an Illumina Novaseq 6000 platform. Q30 score (i.e inferred base call accuracy = 99.9%) was > 80%, average depth 178.5x (range: 135.7-293.3), and average bp % with coverage >20x: 97.13% (range: 96-98.5%). Variants listed in the VCF file were filtered and prioritized based on sequence quality assessment (Q>30; mean coverage >90x; % of bp with coverage>20x >80%); population frequency (minor allele frequency <1% in gnomAD (V2.1.1); variant effect (missense, nonsense, frameshift, splicing effect), in silico pathogenicity prediction (CADD_1.6 score >20; [4], and inheritance pattern, with help of the software package (VarSeq V2.5.1, Golden Helix, MT, USA). BAM files were visualized with help of the Alamut Visual Plus software (V1.6.1, Sophia Genetics SA, Switzerland). Genic and intragenic CNVs were screened with the bioinformatics tool VarSeq CNV Caller (Iacocca et al., 2017), included in the VarSeq (V2.5.1) software (Golden Helix, MT, USA). Variant classification was performed according to ACMG recommendations [1]. A full list of detected variants in our patients is available on request.

**SUPPLEMENTARY CLINICAL INFORMATION**

For all patients, numerical data are presented in **Supplementary Tables 1-4**. For group 2 (subgroups 2a, 2b and 2c), clinical data are presented in the following paragraphs.

**Subgroup 2a: subjects with severe short stature (height <-3.5 SDS) and microcephaly (head circumference <-3.0 SDS) (Supplementary Table S2)**

Patients 13 and 14 from two reportedly unrelated families living in the same city (Gaziantep, Turkey) were homozygous for an identical pathogenic variant in *SMARCAL1*, consistent with the diagnosis of Schimke immuno-osseous dysplasia (MIM #242900). Most of their clinical features were similar as previously reported [5], such as the coarse or fine hair, depressed nasal bridge and bulbous nasal tip, short neck, hypertension, proteinuria, lumbar lordosis and protruding abdomen. Case 13 passed away at 8 years, and his sister with the same clinical presentation (nephrotic syndrome) had died of severe pneumonia at 8 years. Case 14 is on peritoneal dialysis since the age of 4 years. At 9.6 years of age, she suffered from hypertension, numbness in hands and feet, and seizures due to recurrent cerebral strokes. Her height and weight SDS had further decreased (-9.0 and -7.1 SDS, respectively). One of the patient’s cousins was also diagnosed with Schimke immuneosseous dysplasia and died from renal insufficiency and cerebral stroke at 12 years of age. We observed several clinical features in these patients that were not included in the clinical synopsis of the syndrome in OMIM: preterm birth (32 and 28 weeks, respectively); microcephaly (head circumference -3.7 SDS); and serum IGF-I close to the lower limit of the reference range (-1.7 SDS). In contrast to previous observations, body proportions as assessed by sitting height/height ratio were normal (0.6 and -1.4 SDS, respectively). Radiological images of pelvic bones showed bilateral displayed hypoplastic capital femoral epiphyses, without other radiological abnormalities reported in patients with Schimke immuno-osseous dysplasia.

In patient 15 a heterozygous pathogenic *SRCAP* variant was detected, consistent with Floating-Harbor syndrome (MIM #136140). He shared many of the typical clinical characteristics that were described previously for children with this syndrome, including prenatal onset of short stature, triangular face, posteriorly rotated ears, prominent nose, wide columella, smooth philtrum, thin lips, broad mouth, and short neck. Head circumference was initially very low (-3.7 SDS), in line with a few previous reports, including one from Turkey [6, 7]. At follow-up at 7.9 years, height SDS remained -3.7 SDS, but head circumference SDS had increased to -2.4. The patient had a severe speech delay (spoke only ≈50 words, no full sentences). Serum IGF-I was normal. The same variant has been found in six previous reports [8-13].

In 5 patients from families 16-19, homozygosity was shown for four different pathogenic *PCNT* variants (3 of which are novel), with the typical clinical features of microcephalic osteodysplastic primordial dwarfism type 2 (MOPD2, MIM #210720). Case 16 has persistent anaemia and iron deficiency. We have not detected persistent leucocytosis or thrombocytosis in any of these patients.

In patient 20 a novel homozygous *WDR4* variant was detected, associated with a recently reported form of primordial dwarfism, called “microcephaly, growth deficiency, seizures, and brain malformations” (MIGSB, MIM #618346). The clinical features of our patient included a low birth size, seizures from 3 years of age and a severely delayed speech and psychomotor development (she started walking at 5 years). An MRI showed diffuse cerebellar atrophy and small focal signal changes at bilateral frontal subcortical white matter and centrum semi-ovale. These features are similar to previous reports [14-17]. The pituitary had a height of 6.5 mm and volume of 300 mm^3^ (reference 198.92±61.80 mm^3^) [18] at the age of 10.8 years. Serum TSH was 9.77 mU/L, but FT4, FT3, ACTH, cortisol and prolactin were normal. Levothyroxine treatment was started. Interestingly, this patient also showed an extremely low serum IGF-I and a low GH peak (3.68 ng/mL) in a stimulation test (**Supplementary Table S2**).

In patient 21 we found a maternally inherited heterozygous variant of unknown significance (VUS) in *GHSR* in the proband and three short relatives: the mother (-3.3 SDS), a maternal aunt (-2.7 SDS) and one of the proband’s sisters (-2.2 SDS). The variant was not present in relatives with normal stature. The proband’s father has a normal height (-1.0 SDS). The patient presented at 11.8 years with non-dysmorphic short stature (-3.7 SDS), developmental delay and intellectual deficit. At 14.9 years (Tanner stage 3), height SDS had decreased to -4.1 SDS and serum IGF-I and IGFBP-3 had decreased, though the peak GH in a stimulation test was normal. RhGH treatment was administered for 9 months, resulting in a height increment of 9.5 cm and height SDS of -3.4 SDS, while bone age was 2.7 years delayed. Reported adult height was 165 cm (-2.0 SDS). The immature Tanner stage at 14.9 years and the subsequent increase of height in late adolescence from -4.1 to -2.0 is strongly suggestive for delayed puberty. We speculate that the combination of short stature in childhood and delayed puberty in the proband is caused by the maternal *GHSR* variant. Since this variant cannot explain the patient’s microcephaly and intellectual deficit, we expanded the DNA search to these features, and found two novel heterozygous missense variants in *CCND2*, located near each other on the same allele (in cis). Based on the recently reported five cases from three families with a heterozygous loss-of-function *CCND2* variant in the proximal region of the gene, the main effect of such variant is microcephaly, mildly simplified cortical gyral pattern, symmetric borderline short stature, and mild developmental delay [19]. Segregation analysis showed that this variant was also carried by the father. Unfortunately, information on paternal head circumference and intelligence is unavailable. Functional studies are needed to test the hypothesis that the paternal *CCDN2* variants are associated with the patient’s microcephaly and developmental delay.

In patient 22 no genetic cause could be found. He was born small for gestational age (SGA) and presented with severe short stature, microcephaly, developmental delay, mild dysmorphic features (thick eyebrows, prominent eyelashes, short philtrum with low hanging columella, narrow mouth with rather thick lips) and borderline low IGF-I, IGFBP-3 and GH peak. His first-degree consanguineous parents were borderline short (height -2.1 and -1.4 SDS, respectively). Brain MRI showed a small pituitary (160 mm^3^, reference range 300±100 mm^3^). The patient was treated with rhGH but was then lost to follow up. No pathogenic variants were identified in a targeted gene panel nor in microarray analysis.

**Subgroup 2b: Supplementary clinical information on subjects with syndromic short stature (Supplementary Table S3)**

In DNA from patient 23, two 17p13.3 microdeletions separated by a small non-deleted region of 101.5 kb were found. The relatively large distal deletion contains *YWHAE*, *CRK* and 29 other genes, but neither deletion contains *PAFAH1B1*. Similar genetic findings have been reported in 19 individuals with similar clinical features as our patient [20]. The distinctive facial dysmorphism and malformations of cortical development appear to be associated with the deletion of *YWHAE*, but these features are more severe in patients with a larger deletion [21]. Our patient was born extremely SGA (weight -6.1 SDS, length -3.9 SDS), as the oldest of two children of first-degree consanguineous parents. Father’s height was unknown, maternal height was 155 cm (-1.4 SDS). Psychomotor milestones were normal. She presented at 2.9 years with severe short stature, depigmented and sparse hair, frontal bossing and relative macrocephaly and increased sitting height to height ratio (4.0 SDS) associated with rhizomelia. Serum IGF-I and IGFBP-3 were strongly decreased, but GH secretion was normal. Cardiac sonography showed a secundum atrial septum defect and patent ductus arteriosus.

Genetic analysis for patient 24 showed a homozygous pathogenic *TTC37* variant, consistent with Trichohepatoenteric syndrome 1 (THES1, MIM 222470). He presented at 4.2 years with a history of pneumonia at 3 months, frequent diarrhoea until 3 years, lactose intolerance and eczema. Diarrhoea had not responded to the lactose-free diet. Stool frequency was acceptable on a peptide-based formula. We observed 6 renal stones in the right kidney (7 mm diameter). He is the first child of first-degree consanguineous parents (father’s height -1.4 SDS, mother’s height -1.3 SDS). At physical examination, microcephaly, brittle nails and dry discoloured woolly hair were noted. Sitting height/height ratio was 1.3 SDS. Several clinical features of the patient have been associated with the broad spectrum of phenotypic expression in THES1, such as intrauterine growth retardation, woolly hair, facial dysmorphism, intractable diarrhoea in infancy and immunodepression [22, 23]. Microcephaly was noted in a recently reported case [24]. Complete blood count, immunoglobulins and antibody responses were normal, but the CD4 percentage was decreased.

The two siblings in family 25 were included in a paper on 9 families with a novel dysmorphic short stature syndrome (MIM #619184) caused by bi-allelic *SCUBE3* variants. For details regarding clinical information the reader is referred to this publication [25].

In patient 26 we detected a *de novo* heterozygous frameshift variant in *NSD2*. She was born severely small for gestational age. At presentation at the clinic, she was short and microcephalic, and showed mild dysmorphic features (triangular face, periorbital hyperpigmentation, short philtrum and thin elevated nasal bridge). Both parents had a borderline low height (-1.9 SDS). IGF-I was normal. Key features of this syndrome are pre- and postnatal growth delay, failure to thrive, microcephaly, distinctive facial features and normal IGF-I, all consistent with observations in our patient. Most patients display mild cognitive impairment, but some go to a regular school as did our patient. Formal IQ testing was not performed in our patient.

In patient 27 we found a novel homozygous variant in *RABGAP1*, which was recently described as the cause of a neurodevelopmental syndrome in five patients (carrying three different variants) with intellectual disability, microcephaly, bilateral sensorineural hearing loss, seizures and overlapping dysmorphic features [26]. In this publication, two out of five patients were short (-2.0 and -3.0 SDS) and the other three had a height in the lower half of the reference range (-0.4, -0.7 and -0.9 SDS). Since mTOR signalling was downregulated in cells of patients with loss-of-function *RAGGAP1* variants [26], and mTORC1 is a key regulator of cell growth and proliferation and mRNA translation, we postulate that short stature is a (non-obligatory) feature of this novel syndrome. The patient was born with a very low birth weight and presented with psychomotor and speech delay, intellectual deficit and bilateral hearing loss. He first started to walk at 26 months. Parents were healthy and a normal height. Presently, he cannot make sentences and cannot make himself understood. He has not had seizures. Height SDS was similarly decreased as head circumference SDS. Dysmorphic features include a coarse face, short neck, mild upslant of palpebral fissures, prominent eyelashes, upsweep of the lateral part of the eyebrows, wide nasal ridge, mildly low-set ears with overfolded upper part of the helix and underfolded lower part, everted lower lip, short hallux on the left side. Bone age was delayed. Serum IGF-I and IGFBP-3 were in the upper half of the reference range (1.3 SDS) and the GH peak in a stimulation test was normal.

In patient 28, no certain clinical or genetic diagnosis could be made. Information on birth size was missing. Besides short stature, there were no complaints. Physical examination showed normal body proportions and no dysmorphic features (head circumference was not measured). The father was severely short (-3.4 SDS) and mother’s height was in the lower half of the reference range (-1.6 SDS). All three siblings of the patient were short (range -3.9 to -2.9 SDS). Bone age was 1.5 years delayed. Serum IGF-I and IGFBP-3 were low, but the GH response to a stimulation test was normal. A high dose IGF generation test (100 mcg/kg) revealed a normal IGF-I and IGFBP3 response. A SNP-array showed an interstitial duplication of minimally 618.4 kb, containing a large part of one protein coding gene (*SORCS3*), not previously described [arr[hg19], 10q25.1(106,360,639-106,979,017)x3 paternal]. The SNP-array was not performed in the siblings of this patient. Interestingly, we found a homozygous novel VUS in *IHH* NM_002181.4(IHH):c.877A>G p.(Thr293Ala), and both parents were heterozygous carriers of the variant. Homozygous or compound heterozygous pathogenic variants in the IHH gene are associated with Acrocapitofemoral dysplasia (ACFD, (MIM #600726)), a skeletal dysplasia characterized by postnatal-onset disproportionate short stature, relatively large head, narrow thorax, lumbar lordosis, short limbs, and brachydactyly with small broad nails. In-silico prediction of pathogenicity of the identified missense variant is not strong, however this variant is not present in homozygous state and only observed in two alleles in heterozygous state in 278,838 control alleles (GnomAD v2.1.1 database). Additional segregation analysis in three siblings with growth failure showed that the *IHH* variant was present in heterozygous (n=2) and homozygous (n=1) state, while the heights of the siblings were similar (range -2.9 – 3.9 SDS). Based on the in-silico prediction and segregation data we hypothesize that the *IHH* variant does not explain the short stature in this family.

In case 29 we detected a paternally transmitted heterozygous variant of uncertain significance (VUS, PM1, PM2 PP3) in *ACAN* (Chr15(GRCh37): g.89386688A>G, NM_001369268.1:c.860A>G p.Tyr287Cys)), the variant turned out to be present in homozygous state in the father. The more severe short stature in the *ACAN* heterozygous patient (-4.0 SDS) compared with her *ACAN* homozygous father (-2.4 SDS), as well as the presence of multiple other clinical features, are suggestive for another genetic defect that is more consistent with the phenotype. The patient was born SGA as the first of four children from first-degree consanguineous parents. Father’s height is 161.5 cm (-2.4 SDS) and mother’s height 160 cm (-0.5 SDS). No sign of an *ACAN*-related disorder was noticed at visual inspection of the father. Several maternal cousins of the patient are known with short stature with a similar phenotype. The medical history included frequent diarrhoea and otitis in infancy. Psychomotor milestones were normal, but school performance was poor. At physical examination, a broad nasal bridge, retrognathia, mid-facial hypoplasia, short neck, hyperlordosis and a congenital missing fifth toe at the right foot were noted. Body proportions were normal (sitting height/height ratio 0.1 SDS). Serum IGF-I was low but GHD was excluded by a normal GH peak at a GH stimulation test. Radiographic images showed lumbar lordosis and growth arrest lines at the femur and proximal metaphysis of tibia, mild tibial bowing, and gyral impressions at the skull. At 17.7 years she had reached an adult height of 132 cm (-5.3 SDS) with a low BMI (-3.7 SDS). Menarche had occurred at 15 years of age and was followed by oligomenorrhoea. At endocrine testing, serum LH and FSH were normal, as well as other endocrine functions tests. Pelvic ultrasonography showed no abnormality. She is currently on combined oestrogen and progesterone treatment.

In patient 30, no clinical or genetic diagnosis could be made. The patient’s past medical history included a very low birth weight, psychomotor and speech delay, intellectual deficit, recurrent otitis media and bilateral hearing loss. At physical examination, she was extremely short with normal body proportions and borderline microcephaly, thus relative macrocephaly. Dysmorphic features included almond eyes, retrognathia, prominent nose and a thin upper lip. She did not show pubertal signs and bone age was delayed. Biochemical screening showed a low serum IGF-I but normal GH peak in a stimulation test. Genetic testing showed that the patient carried a heterozygous pathogenic variant in *LARP7* (NM_016648.4(LARP7):c.475_478dup, p.Lys160Ilefs*2), but this variant was also present in the normal statured mother (height 0.5 SDS).

In patient 31, no clinical or genetic diagnosis could be made. Past medical history included a low birth weight, bilateral hearing loss (noticed at 1 year of age), delayed psychomotor and speech development and subnormal cognitive development. A hearing device was administered at 3.4 years. He was short and mildly microcephalic. Dysmorphic features included mildly triangular face, bushy eyebrows, low hanging columella, low-set ears, and mild micrognathia. There was a severe hyperlordosis. Parents are healthy and of normal height. Serum IGF-I and IGFBP-3 were within the reference range. A novel homozygous variant of unknown significance in *SPATA5* was found: Chr4(GRCh37):g.123850301A>G, NM_145207.3: c.395A>G p.(Gln132Arg) (ACMG: PM1, PM2, BP4). The patient presented with several clinical features that seem consistent with “neurodevelopmental disorder with hearing loss, seizures, and brain abnormalities” (NEDHSB, MIM #616577), caused by bi-allelic *SPATA5* variants. These features include short stature, developmental delay, microcephaly, sensorineural hearing loss, low birth size, low set ears and broad/thick eyebrows. Several clinical features of the patient, including poor growth and facial Gestalt, are similar to the cases 2 and 3 reported by Kurata et al [27] and a patient published by Buchert et al [28]. However, the results of the *in silico* prediction tools of the *SPATA5* variant leaves us in doubt whether this genetic finding is associated with the clinical presentation. Functional studies of this variant are needed to confirm our speculation.

**Subgroup 2c: Subjects with growth hormone deficiency**

Numerical data of the 3 patients with GHD are shown in **Supplementary Table 4**. The results of the WES analysis were non-conclusive.

Patient 32 was born with a normal birth weight and besides severe proportionate short stature there were no complaints. Head circumference was in the lower half of the reference range. Paternal and maternal heights were -2.1 and -2.8 SDS. Serum IGF-I and IGFBP-3 were low. The GH peaks in two stimulation tests was were 3.3 ng/mL (clonidine) and 6.8 ng/mL (glucagon). An IGF generation test showed a positive response. Other pituitary hormones were normal and a brain MRI showed a normal pituitary. Candidate gene testing for *GH1, GHRHR* and *GHSR* showed normal results, and WES results were inconclusive. RhGH treatment was started at 6 years of age. On treatment, the first-year height velocity was 7.5 cm, but compliance appeared poor. The patient was then lost to follow up.

Patient 33 was born with a borderline low weight. The medical history was uneventful. Height was short, head circumference normal, and sitting height/height ratio elevated (3.2 SDS). No dysmorphic features were noted. Paternal and maternal heights were -2.1 and -1.5 SDS. Serum IGF-I was in the lowest half of the reference range, but serum IGFBP-3 was very low. The GH peaks in stimulation tests were 0.6 ng/mL (glucagon) and 2.2 ng/mL (clonidine). The brain MRI was normal. Trio WES analysis did not yield a positive result.

Patient 34 was born with a normal weight. The medical history was uneventful except for widespread severe eczema, cheilitis, peeling skin, onychomycosis and acral punctate keratosis starting at 1 year of age. Height was proportionately short and head circumference was borderline low. Besides his skin phenotype, physical examination showed curly hair, a relatively small face, and coarse facial features. Paternal and maternal heights are -0.8 and -1.7 SDS. Serum IGF-I and IGFBP-3 were low and the GH peak in stimulation tests were 0.2 ng/mL (glucagon) and 5.5 ng/mL (clonidine). MRI showed a small adenohypophysis, small pituitary stalk and ectopic neurohypophysis. The clinical features suggested Netherton syndrome, but no pathogenic variant was found in *SPINK5*. However, a homozygous likely pathogenic nonsense variant in *CAST* exon 10: Chr5(GRCh38):g.96740074A>T, NM_001042440.5: c.712A>T p.(Lys238*), (ACMG: PVS1, PM2) was found by WES analysis of the proband and his mother [29], which we consider to be the cause of his skin phenotype.

Supplementary Table S1. Group 1; Clinical and biochemical features and candidate gene analysis in subjects with pathogenic variants in the GH-IGF-I axis

| **Patient number** | **Sex** | **Age (yrs)** | **BA delay (yrs)** | **BW**  **SDS** | **Height SDS** | **HC SDS** | **BMI SDS** | **IGF-I**  **SDS** | **IGFBP-3 SDS** | **GH basal (ng/mL)** | **GH max**  **(ng/mL)** | **IGF gen test (ng/mL)** | **Clinical features** | **Genetic findins (all homozygous)** |
| --- | --- | --- | --- | --- | --- | --- | --- | --- | --- | --- | --- | --- | --- | --- |
|  |  |  |  |  |  |  |  |  |  |  |  |  |  | *GH1* gene |
| 1a | M | 4.5 | 1.75 | 1.1 | -4.6 | -1.3 | -4.6 | <-3.0 | <-3.0 | <0.05 | <0.05 | nd | Classical,  hypoglycaemia | Deletion of *GH1* exon 3, 4 and 5^b^ |
| 1b | F | 3.2 | 1.66 | 2.0 | -8.5 | -4.0 | -0.8 | <-3.0 | <-3.0 | <0.05 | <0.05 | nd | Classical,  hypoglycaemia |  |
|  |  |  |  |  |  |  |  |  |  |  |  |  |  | *GHR* gene: |
| 2 | M | 10.1 | 2.2 | -0.2 | -4.3 | -1.1 | -0.7 | <-2.8 | <-2.6 | 1.8-1.9 | 20.0 | No resp | Dry skin, orchidopexy | c.344A>C  p.Asn115Thr |
| 3a | F | 5.0 | 2.0 | 0.5 | -3.8 | -1.6 | -0.4 | -2.2 | -2.8 | 13.3 | nd | +25 | Classical | c.344A>C  p.Asn115Thr |
| 3b | F | 10.7 | na | na | -2.4 | -2.5 | -0.7 | -2.6 | -3.1 | 0.7 | nd | nd | Classical |  |
| 3c | M | 8.5 | 1.5 | na | -3.3 | -1.5 | 1.0 | -3.2 | -3.7 | 0.8 | nd | nd | Classical |  |
| 4a | M | 7.9 | 4.3 | -1.2 | -3.9 | -2.0 | -1.7 | <-3.9 | -3.0 | 1.5 | 12.1 | No resp | Classical | c.908T>A  p.Val303Asp |
| 4b | M | 4.0 | 1.5 | -1.4 | -2.1 | -1.6 | -1.0 | -2.9 | -1 | 1.4 | nd | nd | Classical |  |
| 5a | F | 6.5 | 3.75 | 0.3 | -7.2 | na | 0.9 | <-3.0 | <-3.0 | 40 | nd | No resp | Classical | c.173C>T  p.Ser58Leu |
| 5b | F | 16.5 | 6.0 | -0.2 | -11.2 | na | -1.4 | <-3.0 | <-3.0 | 3.3, 8.7 | 40 | No resp | Classical |  |
| 6 | M | 2.6 | na | -0.8 | -3.9 | na | 0.7 | <-3.0 | <-3.0 | na | 17.7 | No resp | Hypothyroidism, sensorineural deafness | c.618+792A>G |
|  |  |  |  |  |  |  |  |  |  |  |  |  |  | *STAT5B* gene: |
| 7 | F | 17.6 | 5.2 (at 15.2) | -1.6 | -5.2 | -2.0 | 1.9 | undet | <-3.0 | 0.7 | 3.8 | nd | Eczema, midline hypoplasia, frontal bossing, obesity, PRL↑ | c.1453delG p.Asp485Thrfs*29 |
|  |  |  |  |  |  |  |  |  |  |  |  |  |  | *IGFALS* gene: |
| 8a (A.III.5^a^) | M | 8.0 | 2.4 | -1.3 | -3.7 | -2.9 | -0.3 | -4.5 | -9.1 | 3.8 | nd | No resp | none | c.1462G>A  p.Asp488Asn |
| 8b (A.III.14^a^) | M | 11.5 | 0.4 | na | -3.7 | -2.8 | -0.5 | -3.6 | -10.2 | 11.9 | 12.9 | +29 | none |  |
| 9 (B.IV.2^a^) | M | 9.4 | 3.0 | -2.6 | -2.0 | -2.0 | -1.2 | -3.3 | -11.2 | 0.6 | 26.4 | nd | none | c.1462G>A  p.Asp488Asn |
| 10 (C.V.5^a^) | F | 13.5 | 2.5 | -3.8 | -3.6 | -2.1 | -1.1 | -3.2 | -8.1 | 0.4 | 8.2 | nd | none | c.251A>G  p.Asn84Ser |
| 11 (D.IV.3^a^) | M | 11.4 | 2.0 | na | -2.3 | -2.2 | -1.6 | -3.8 | -10.0 | na | 10.3 | No resp | none | c.1477del  p.Arg493Alafs*176 |
| 12 (E.IV.2^a^) | F | 11.8 | -0.5 | -2.4 | -3.7 | -1.9 | -0.1 | -3.0 | -6.5 | 2,1 | 12 | +21 | none | c.251A>G,  p.Asn84Ser/  c.1462G>A, p.Asp488Asn |

Abbreviations: BA, bone age, BW, birth weight;. hc: head circumference; IGF gen test, IGF generation test; na, not available; Nd, not done; PRL, prolactin; resp, response; undet, undetectable;

^a^codes as published in Isik et al [30]. ^b^GH1 NC_000017.10:g.(?_61995432)_(61994614 _?)del, a deletion of *GH1* exon 3, 4 and 5 detected with a MLPA assay (MRC-Holland kit P216) where the nucleotide positions g.61995433 and g.61994615 are defined by the first nucleotide of the probe 3’- of the ligation site for respectively exon 3 and exon 5.

Supplementary Table S2. Subgroup 2a; Clinical and biochemical features and genetic findings in subjects with microcephaly (head circumference <-3.0 SDS) and severe short stature (height <-3.5 SDS)

| **Patient number** | **Sex** | **Age (yrs)** | **BA delay (yrs)** | **BW**  **SDS** | **Height SDS** | **HC SDS** | **BMI SDS** | **IGF-1**  **SDS** | **IGFBP-3 SDS** | **GH basal (ng/mL)** | **GH max**  **(ng/mL)** | **IGF gen test (ng/mL)** | **Clinical features** | **Genetic findings** |
| --- | --- | --- | --- | --- | --- | --- | --- | --- | --- | --- | --- | --- | --- | --- |
| 13 | M | 5.9 | 2.3 | -4.1 | -6.7 | -3.7 | 0.0 | -1.7 | -4.4 | 8.0 | 13.4 | nd | Abnormal hair, depressed nasal bridge, bulbous nasal tip, lumbar lordosis, proteinuria, hypertension. Deceased at 8 yrs. | Hom *SMARCAL1* variant,  NM_014140.4: c.2459G>A p.Arg820His |
| 14 | F | 1.9 | 0.5 | -0.4 | -5.2 | -3.7 | -1.3 | -3.1 | -0.6 | 2.0 | 5.6 | nd | Fine hair, thin upper lip, bulbous nasal tip, lumbar lordosis, FSGS, end stage renal disease, developmental delay, shallow acetabular fossa | Hom *SMARCAL1* variant,  NM_014140.4: c.2459G>A p.Arg820His |
| 15 | M | 0.9  7.9 | na | -2.0 | -3.7  -3.7 | -3.7  -2.4 | -2.5  -0.4 | 0.1 | 0.6 | 13.7 | nd | nd | Triangular face, thin and horizontal lips, high pitched voice, microcephaly | Het *SRCAP* variant,  NM_006662.3: c.7303C>T p.Arg2435* |
| 16 | M | 0.9 | na | -5.6 | -15.5 | -10.6 | -4.6 | -2.7 | -0.4 | 14.6 | nd | nd | Primordial dwarfism, frequent infections, developmental delay | Hom *PCNT* variant  c.5180dupA p.Asn1727LysfsTer14 |
| 17a | M | 10.0 | na | -4.5 | -8.7 | -10.9 | -4.2 | 2.3 | -0.5 | 0.1 | nd | nd | Prominent nose, micrognathia, microdontia, hypo- and hyperpigmentation, cutis marmorata, café-au-lait spots, curved fingers, developmental delay | Hom *PCNT* variant  NM_006031.6: c.3109G>T p.Glu1037* |
| 17b | F | 0.75 | na | -5.5 | -7.8 | -8.3 | -1.7 | 3.8 | 1.2 | 14 | nd | nd | Primordial dwarfism |  |
| 18 | M | 10.9 | na | -4.4 | -6.3 | -6.5 | -3.3 | -0.2 | -0.7 | 1.2 | 24.5 | nd | Primordial dwarfism | Hom *PCNT* variant  NM_006031.6: c.721-2A>G p.? |
| 19 | F | 4.3 | 2.5 | 500g (26wks) | -8.5 | -8.4 | -4.7 | -0.7 | 0.5 | nd | nd | nd | Developmental delay, scoliosis, partial empty sella | Hom *PCNT* variant  NM_006031.6: c.3608_3840del p.Pro1204Glyfs*11,  Confirmed at RNA level |
| 20 | F | 9.7 | 1.9 | -2.4 | -5.9 | -4.5 | -1.3 | -5.2 | -1.3 | 0.2, 1.1, 3.9 | 3.86 | nd | Developmental delay, does not talk, convulsions, mental retardation, ataxia, walking with support | Hom *WDR4* variant  NM_018669.6: c.428G>A p.Gly143Glu |
| 21 | M | 11.8  14.9  15.718.0 | 2.7 | Low | -3.7  -4.1  -3.4  -2.0 | -3.9 | -2.2 | -0.6,  -1.5 | -0.5,  <-2.0 |  | 21 | nd | Developmental delay (IQ 78) | Het *GHSR* variant (VUS)  NM_198407.2: c.1049C>G p.Thr350Ser |
| 22 | M | 9.2 | 3.0 | -4.4 | -4.1 | -5.4 | -2.4 | -1.9 | -1.7 | na | 6.3 | nd | Developmental delay, small pituitary | No causal variant |

Abbreviations: BA, bone age; BW, birth weight; FSGS: focal segmental glomerulosclerosis. hc, head circumference; Het, heterozygous; Homozyg, homozygous; IGF gen test: IGF generation test; na, not available; nd, not done; resp, response

Supplementary Table S3. Subgroup 2b; Clinical and biochemical features and genetic findings in subjects with syndromic short stature

| **Patient number** | **Sex** | **Age (yrs)** | **BA delay (yrs)** | **BW**  **SDS** | **Height SDS** | **HC SDS** | **BMI SDS** | **IGF-I**  **SDS** | **IGFBP-3 SDS** | **GH basal (ng/mL)** | **GH max**  **(ng/mL)** | **IGF gen test (ng/mL)** | **Clinical features** | **Genetic findings** |
| --- | --- | --- | --- | --- | --- | --- | --- | --- | --- | --- | --- | --- | --- | --- |
| 23 | F | 2.9 | 1.6 | -6.1 | -5.2 | -1.3 | 0.5 | <-2.0 | -4.3 | 3.5, 2.0 | 20 | No response | Rhizomelia, frontal bossing, hair depigmented and sparse, atrial septum defect, patent ductus arteriosus | 17p13.3 microdeletion  (525-1,922,715)x1, (2,024,217-2,262,703)x1  Arr[hg19] |
| 24 | M | 12.4 | 2.0 | -1.7 | -3.3 | -2.8 | -2.1 | -0.7 | -3.0 | 1.2 | 11.7 | nd | Diarrhoea, eczema, dry discoloured hair | Hom *TTC37* variant  c.4572G>A, p.Trp1524* |
| 25a | M | 13.0 | 0.9 | -2.6 | -3.6 | -3.7 | -2.6 | -1.2 | 0.5 | 1.6 | 17 | nd | Frequent infections, triangular face | Hom *SCUBE3* variant  c.2599+2T>C p.?^a^ |
| 25b | F | 8.5 | 0.7 | -0.4 | -3.9 | -1.9 | -1.4 | -0.4 | 0.6 | 0.6 | 9.4 | nd | Frequent infections, triangular face, idiopathic frequent ventricular extrasystoles |  |
| 26 | F | 8.5 | 0.7 | -3.9 | -3.1 | -3.2 | -2.3 | -1.2 | 1.5 | 8.4 | nd | nd | Triangular face, periorbital hyperpigmentation, short philtrum, thin elevated nasal bridge. | Het *NSD2* variant  c.642dup, p.Asp215Argfs*10 |
| 27 | M | 8.8 | 3.0 | -3.8 | -3.3 | -3.2 | -0.5 | 1.4 | 1.3 | 0.3 | 11.2 | nd | Intellectual delay, speech delay, bilateral hearing loss | Hom *RABGAP1* variant  c.2789dup  p.Asn930Lysfs*7 |
| 28 | M | 12.5 | 1.4 | na | -4.1 | -1.5 | -1.1 | -2.1 | -3.6 | na | 22 | +390 on high GH dose | none | No causal variant |
| 29 | F | 17.7 | 0.6 | -2.6 | -5.3 | -2.5 | -3.7 | -2.1 | -0.7 | 0.5 | 17.2 | nd | Frequent infections, developmental delay, absent toe, wide nasal bridge, retrognathia, hyperlordosis | No causal variant |
| 30 | F | 13.5 | 3.0 | -3.8 | -7.0 | -2.2 | -3.8 | -3.0 | 1.3 | 0.14 | 13.1 | nd | Almond eyes, retrognathia, prominent nose, thin upper lip, absent pubertal signs | No causal variant |
| 31 | M | 10.0 | 4.0 | -2.8 | -3.8 | -2.5 | -1.5 | -0.9 | 1.1 | 0.4 | 7.0 | nd | Bilateral hearing loss, developmental delay, triangular face, hyperlordosis, low-set ears | No causal variant |

Abbreviations: BA, bone age; BW, birth weight; hc, head circumference; Het, heterozygous; Homozyg, homozygous; IGF gen test, IGF generation test; na, not available; nd, not done;

^a^c.2599+2T>C is predicted to result in multiple aberrantly processed transcripts, with p.Asn801Thrfs∗127 representing the prevalent out-of-frame product [25].

Supplementary Table 4. Subgroup 2c; Clinical, biochemical and genetic features of subjects with nonspecific growth hormone deficiency

| **Patient number** | **Sex** | **Age (yrs)** | **BA delay (yrs)** | **BW**  **SDS** | **Height SDS** | **HC SDS** | **BMI SDS** | **IGF-I**  **SDS** | **IGFBP-3 SDS** | **GH basal (ng/mL)** | **GH max**  **(ng/mL)** | **IGF gen test (ng/mL)** | **Clinical features** | **Genetic findings** |
| --- | --- | --- | --- | --- | --- | --- | --- | --- | --- | --- | --- | --- | --- | --- |
| 32 | M | 3.75 | 1.5 (at 4.8 yrs) | 0.4 | -4.7 | -1.6 | 0.0 | <-2 | -2.6 | 1.0 | 6.8, 3.3 | >15.5 | Poor response to rhGH, possible partly to poor adherence. | No causal variant |
| 33 | M | 7.3 | 3.5 | -1.9 | -4.5 | -0.8 | 1.4 | -1.5 | -2.9 | 0.4 | 2.2, 0.6 | nd | Good response to rhGH | No causal variant |
| 34 | M | 2.8 | 0.8 | -1.7 | -3.8 | -2.1 | -0.1 | <-1.7 | -1.4 | 0.1 | 5.5, 0.23 | Normal IGF-I and IGFBP-3 after 3 m rhGH | Eczema, curly hair, candidiasis of toenails. MRI: small adenohypophysis, small pituitary stalk, ectopic neurohypophysis | No causal variant |

Abbreviations: BA, bone age; Nd, not done; m, months; rhGH, recombinant human growth hormone

**References**

1 Richards S, Aziz N, Bale S, Bick D, Das S, Gastier-Foster J, et al.: Standards and guidelines for the interpretation of sequence variants: a joint consensus recommendation of the American College of Medical Genetics and Genomics and the Association for Molecular Pathology. Genet Med 2015;17:405-424.

2 Biesecker LG, Harrison SM, ClinGen Sequence Variant Interpretation Working G: The ACMG/AMP reputable source criteria for the interpretation of sequence variants. Genet Med 2018;20:1687-1688.

3 Griffith E, Walker S, Martin CA, Vagnarelli P, Stiff T, Vernay B, et al.: Mutations in pericentrin cause Seckel syndrome with defective ATR-dependent DNA damage signaling. Nat Genet 2008;40:232-236.

4 Kircher M, Witten DM, Jain P, O'Roak BJ, Cooper GM, Shendure J: A general framework for estimating the relative pathogenicity of human genetic variants. Nat Genet 2014;46:310-315.

5 Elizondo LI, Cho KS, Zhang W, Yan J, Huang C, Huang Y, et al.: Schimke immuno-osseous dysplasia: SMARCAL1 loss-of-function and phenotypic correlation. J Med Genet 2009;46:49-59.

6 Ioan DM, Fryns JP: Floating-Harbor syndrome in two sisters: autosomal recessive inheritance or germinal mosaicism? Genet Couns 2003;14:431-433.

7 Karaer K, Karaoguz MY, Ergun MA, Yesilkaya E, Bideci A, Percin EF: Floating-Harbor syndrome: a first female Turkish patient? Genet Couns 2006;17:465-468.

8 Hood RL, Lines MA, Nikkel SM, Schwartzentruber J, Beaulieu C, Nowaczyk MJ, et al.: Mutations in SRCAP, encoding SNF2-related CREBBP activator protein, cause Floating-Harbor syndrome. Am J Hum Genet 2012;90:308-313.

9 Ko J, Pomerantz JH, Perry H, Shieh JT, Slavotinek AM, Oberoi S, et al.: Case Report of Floating-Harbor Syndrome With Bilateral Cleft Lip. Cleft Palate Craniofac J 2020;57:132-136.

10 Reschen M, Kini U, Hood RL, Boycott KM, Hurst J, O'Callaghan CA: Floating-Harbor syndrome and polycystic kidneys associated with SRCAP mutation. Am J Med Genet A 2012;158A:3196-3200.

11 Seifert W, Meinecke P, Kruger G, Rossier E, Heinritz W, Wusthof A, et al.: Expanded spectrum of exon 33 and 34 mutations in SRCAP and follow-up in patients with Floating-Harbor syndrome. BMC Med Genet 2014;15:127.

12 Yagi H, Takagi M, Narumi S, Hasegawa T, Nishimura G, Hasegawa Y: Stippled calcification in an infant with a recurrent SRCAP gene mutation. Am J Med Genet A 2016;170A:1088-1091.

13 Zhang S, Chen S, Qin H, Yuan H, Pi Y, Yang Y, et al.: Novel genotypes and phenotypes among Chinese patients with Floating-Harbor syndrome. Orphanet J Rare Dis 2019;14:144.

14 Shaheen R, Abdel-Salam GM, Guy MP, Alomar R, Abdel-Hamid MS, Afifi HH, et al.: Mutation in WDR4 impairs tRNA m(7)G46 methylation and causes a distinct form of microcephalic primordial dwarfism. Genome Biol 2015;16:210.

15 Trimouille A, Lasseaux E, Barat P, Deiller C, Drunat S, Rooryck C, et al.: Further delineation of the phenotype caused by biallelic variants in the WDR4 gene. Clin Genet 2018;93:374-377.

16 Chen X, Gao Y, Yang L, Wu B, Dong X, Liu B, et al.: Speech and language delay in a patient with WDR4 mutations. Eur J Med Genet 2018;61:468-472.

17 Braun DA, Shril S, Sinha A, Schneider R, Tan W, Ashraf S, et al.: Mutations in WDR4 as a new cause of Galloway-Mowat syndrome. Am J Med Genet A 2018;176:2460-2465.

18 Sari S, Sari E, Akgun V, Ozcan E, Ince S, Saldir M, et al.: Measures of pituitary gland and stalk: from neonate to adolescence. J Pediatr Endocrinol Metab 2014;27:1071-1076.

19 Pirozzi F, Lee B, Horsley N, Burkardt DD, Dobyns WB, Graham JM, Jr., et al.: Proximal variants in CCND2 associated with microcephaly, short stature, and developmental delay: A case series and review of inverse brain growth phenotypes. Am J Med Genet A 2021;185:2719-2738.

20 Baker EK, Brewer CJ, Ferreira L, Schapiro M, Tenney J, Wied HM, et al.: Further expansion and confirmation of phenotype in rare loss of YWHAE gene distinct from Miller-Dieker syndrome. Am J Med Genet A 2023;191:526-539.

21 Denomme-Pichon AS, Collins SC, Bruel AL, Mikhaleva A, Wagner C, Vancollie VE, et al.: YWHAE loss of function causes a rare neurodevelopmental disease with brain abnormalities in human and mouse. Genet Med 2023:100835.

22 Fabre A, Martinez-Vinson C, Roquelaure B, Missirian C, Andre N, Breton A, et al.: Novel mutations in TTC37 associated with tricho-hepato-enteric syndrome. Hum Mutat 2011;32:277-281.

23 Bourgeois P, Esteve C, Chaix C, Beroud C, Levy N, consortium Tc, et al.: Tricho-Hepato-Enteric Syndrome mutation update: Mutations spectrum of TTC37 and SKIV2L, clinical analysis and future prospects. Hum Mutat 2018;39:774-789.

24 Gao J, Hu X, Hu W, Sun X, Chen L: Novel TTC37 mutations in a patient with Trichohepatoenteric syndrome: a case report and literature review. Transl Pediatr 2022;11:1050-1057.

25 Lin YC, Niceta M, Muto V, Vona B, Pagnamenta AT, Maroofian R, et al.: SCUBE3 loss-of-function causes a recognizable recessive developmental disorder due to defective bone morphogenetic protein signaling. Am J Hum Genet 2021;108:115-133.

26 Oh RY, Deshwar AR, Marwaha A, Sabha N, Tropak M, Hou H, et al.: Biallelic loss-of-function variants in RABGAP1 cause a novel neurodevelopmental syndrome. Genet Med 2022;24:2399-2407.

27 Kurata H, Terashima H, Nakashima M, Okazaki T, Matsumura W, Ohno K, et al.: Characterization of SPATA5-related encephalopathy in early childhood. Clin Genet 2016;90:437-444.

28 Buchert R, Nesbitt AI, Tawamie H, Krantz ID, Medne L, Helbig I, et al.: SPATA5 mutations cause a distinct autosomal recessive phenotype of intellectual disability, hypotonia and hearing loss. Orphanet J Rare Dis 2016;11:130.

29 Lin Z, Zhao J, Nitoiu D, Scott CA, Plagnol V, Smith FJ, et al.: Loss-of-function mutations in CAST cause peeling skin, leukonychia, acral punctate keratoses, cheilitis, and knuckle pads. Am J Hum Genet 2015;96:440-447.

30 Isik E, Haliloglu B, van Doorn J, Demirbilek H, Scheltinga SA, Losekoot M, et al.: Clinical and biochemical characteristics and bone mineral density of homozygous, compound heterozygous and heterozygous carriers of three novel IGFALS mutations. Eur J Endocrinol 2017;176:657-667.
